# Knowledge, attitude and practices study of acaricide usage and tick control in South Omo Zone pastoral areas, South-Western Ethiopia

**DOI:** 10.1016/j.heliyon.2023.e17212

**Published:** 2023-06-12

**Authors:** Tegegn Tesfaye, Aschenaki Abate

**Affiliations:** Southern Agricultural Research Institute (SARI), Jinka Agricultural Research Center (JARC), Ethiopia

**Keywords:** Acaricide, Acaricide usage, Knowledge and attitude, Herdsmen, Bena-Tsemay, South Omo, Ethiopia

## Abstract

Although acaricide chemotherapy is widely used to control tick infestation in Ethiopia, its effectiveness is uncertain due to misusage by herdsmen. Currently, there is no study being conducted in the South Omo Zone of Ethiopia which shows the knowledge, attitude, and practice (KAP) and associated factors of acaricide usage by herdsmen. Therefore, this study was conducted to assess KAP of 120 (83 male and 37 female) pastoralist and agro-pastoralist of Bena-Tsemay district through structured questionnaire survey. Accordingly, Ivermectin was the most preferred acaricide by majority (62.5%) of the herdsmen. Half (50%) of the herdsmen confessed that price of acaricide is the defining variable for acaricide preference in their location where 60.83% of them obtain acaricides from private drug shops. Majority (60%) of the respondents said that they obtain information about acaricide usage from drug sellers in the vet drug shops. According to 72.50% of the respondents, acaricide application/injection on the infested herd was conducted by the herdsmen. A 95.83% of our interviewee revealed that there was no training or awareness creation being given on how to inject or apply acaricide on tick infested animals. Moreover, all responders (100%) confessed that they didn't have a practice of weighing animals and measuring acaricide dosage prior to injection/application. The incidence of acaricide poisoning on animal and personnel was reported by 19.17% and 22.5% of respondents, respectively. Simple logistic regression analysis revealed that gender (OR = 5.09, OR 95% CI = 2.30–11.72), practice of acaricide rotation (OR = 3.22, OR 95% CI = 1.41–7.64) and personnel preference for acaricide application (OR = 2.66, OR 95% CI = 1.18–6.15) were significantly (P < 0.05) associated with the knowledge score of the respondents. On the other hand, respondent's attitude score was significantly (P < 0.05) associated with their acaricide rotation practice (OR = 3.20, OR 95% CI = 1.39–7.53) and personnel preference for acaricide application (OR = 6.61, OR 95% CI = 2.78–16.93). Similarly, practice of acaricide rotation (OR = 5.31, OR 95% CI = 2.26–12.96) and personnel preference for acaricide application (OR = 7.21, OR 95% CI = 3.03–17.99) were significantly linked with the practice score of the respondents towards acaricide usage. In conclusion, ticks are the major challenge in the study area despite widespread usage of acaricides. Because of extensive misusage of available acaricides, awareness creation should be applied to narrow KAP gaps and to conserve the efficacy of these chemicals. Furthermore, acaricide efficacy investigation (in vitro and in vivo) should be conducted to know the status of commonly used acaricides in the area.

## 1 Introduction

Tick infestation and its effect is one of the main issues restricting livestock productivity that cattle farming communities face, particularly in the tropics and subtropics. 80% of cattle around the world are afflicted by ticks and the diseases they carry. The most important species of cattle ticks and diseases they transmit in Africa are *Rhipicephalus (Boophilus) decoloratus* and *Rhipicephalus (Boophilus) microplus*, which transmit the pathogens causing babesiosis and anaplasmosis; *Rhipicephalus appendiculatus* which transmits *Theileria parva* (the cause of East Coast fever); and species in the genus Amblyomma (particularly *Am. variegatum*) are responsible for transmission of *Ehrlichia ruminantium* which cause heart water [1]. Ticks kill livestock through tick-borne diseases (TBDs); resulting in direct and indirect economic losses. Ticks feeding at the point of attachment cause wounds that degrade the quality of the hide and skin as well as udder damage that causes mastitis [2,3].

Although methods including manual plucking, pasture management and vaccination have also been used in some parts of the world, chemotherapy with direct application of acaricides to host animals continues to be the most popular form of tick control [2,4]. For the control of ticks, a variety of tickicidal compounds have been applied. Organophosphates, synthetic pyrethroids, and amidines are the three main categories of acaricidal pesticides being utilized in Africa. However, some of these compounds are no longer effective in many ticks endemic locations since some tick species have developed resistance to them [5,6]. Along with the emergence of acaricide-resistant tick species, high price, detrimental effects on unintended species, and accumulation of acaricide residues in animal products all preclude their use on animals.

In Ethiopia, ticks are the most prevalent external parasite that infests animals, and their infestation results in considerable financial losses for the livestock industry [7]. The only method for effectively controlling tick parasitosis in Ethiopia is the use of acaricide. In the past, Ethiopian herdsmen have utilized a variety of chemical acaricides to control the tick population, including organochlorines, organophosphates, macrocyclic lactones, carbamates, amidines, and synthetic pyrethroids. However, the misuse of powerful acaricides or the emergence of resistance to already-used classes currently prevents the intended outcomes from being obtained.

Effective tick management with acaricides requires knowledge of ticks, acaricide chemical groups, and adequate acaricide application by the animal owner. Knowing the ideal acaricide-to-water ratio is crucial to achieve the required active ingredient concentration for tick control. These application instructions are listed on the information labels for the various acaricide products. It is unknown, though, whether and how livestock owners will read, comprehend, and apply this information, and how this might affect how well tick treatments function. During our pastoral villages visit in the South Omo Zone, acaricide usage malpractices are widespread in the area. Therefore, to be effective in tick control practice, to anticipate the intended result and to conserve the efficacy of available acaricides, understanding the knowledge and awareness of herders about acaricide usage is very significant. This study was designed to meet its objectives by assessing the knowledge, attitude, and practice (KAP) of pastoralists and agro-pastoralists about acaricide chemical usage and tick management in South Omo zone, South-Western Ethiopia.

## 2 Materials and methods

### 2.1 Study area

**Fig. 1:**
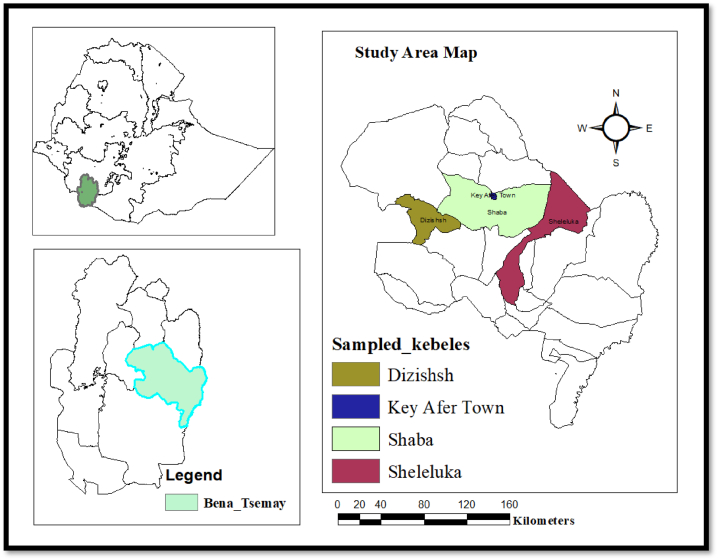
Map of study area: South Omo (left, lower) and Bena-Tsemay district with study villages (right).

The study was conducted in the Bena-Tsemay district (Fig. 1, left lower) in the South Omo zone of Southwest Ethiopia. For this particular study, four pastoral villages (Fig. 1, right); Sheleluka (Luka), Dizishsh (Diziaman), Shaba Argamenda, and Key Afer town (Olkakbo) were arbitrarily chosen from the district. The district is situated between 04° 59.00″ and 05° 58.40″ N, with a total area of 2923 km^2^ [8]. The climate in the Bena-Tsemay district ranges from hot to desert. Temperatures in the area often range between 15.6 and 26.5° Celsius on a daily average. The altitude of the district is 500–1800 m above sea level. It is well known that the Bena-Tsemay district experiences erratic rainfall patterns, which are typically bimodal and fall between September and December and March and May. 800–1300 mm of rain precipitation averages annually in the area [9].

### 2.2 Study population, design and sampling criterion

Participants of this study were the herd owners (pastoralists and agro-pastoralists) residing in Bena-Tsemay district. Cross-sectional questionnaire survey was employed to collect the required information. The main criteria employed to select the respondents were cattle ownership, willingness to participate in the interview and their experience in acaricide usage. All residents who owned cattle were included in the primary sampling frame. Then, herders with willingness to participate and acaricide usage practices were selected from the primary sampling frame to form secondary sampling frame. Finally, random selection was performed to select the required number of respondents from secondary sampling frame.

All the respondents were allowed to test for KAP of acaricide usage and tick control based on pre-set piece of criterion. The criterion used for individuals knowledge (good versus poor) test were; years (>10 years) of experience on acaricide usage, having training on acaricide usage, practice of measuring acaricide dosage and animals body weight prior to deworming, practice of purchasing acaricides from authorized sources and having awareness on possible harm of acaricide on animals and environment. Herd owners with higher desire to participate on tick eradication program and using all possible options (conventional acaricide and ethno-veterinary practices) to control tick were classified as having positive attitude and reluctant ones were classified as having negative attitude. Moreover, for practice (good versus poor); practice of purchasing acaricides from authorized veterinary drug sources, practice of rotating acaricide, practice of measuring acaricide dosage, allowing trained veterinarian/CAHWs to apply/inject acaricide on their herds and using protective clothes during acaricide were used as criterion.

### 2.3 Sample size determination

Sample size estimation formulae of n = $\frac{Z^{2}P\left(1-P\right)}{e^{2}}$, was applied to determine appropriate number of study participants; where z (at 95% confidence level) = 1.96, n = required number of participant, p = estimated baseline proportion of cattle owner who were presumed to have adequate KAP about ticks and its impact on cattle production and acaricide usage practices and e = margin of error = 0.05. According to Bena-Tsemay district livestock expert's (veterinarians, breeding experts, vet clinic practitioners and village level community animal health workers) assumption, only 10% (P = 0.1) of pastoral and agro-pastoral community have adequate KAP about tick and its impact and usage of acaricide products. According to the above formulae, a total of 138 households were required to participate; however, due to various challenges faced during our interview process (displacement of households due to natural calamities, mobile nature of pastoralists and unwillingness of individuals to participate in the study), only 120 household were participated in this study. From each four randomly selected villages, 30 households/pastoral herd owners were recruited in to questionnaire interview.

### 2.4 Data collection

Prior to dissemination of the questionnaire to study participants, the whole questionnaire with sub-sections (dealing knowledge, attitude and practice) was validated through pretesting in a total of 15 purposively selected pastoralists and agro-pastoralists target study area. Then, the validated version of questionnaire was translated in to local dialect (Bena language) before being sent to appropriate participants. Then, the questionnaire was delivered to an interviewee with local dialect with the help of trained translator and the response was recorded in hard copy as well as voice recorded for further revision. In addition to primary information, secondary data sources from the study district were noted and examined. In general, the questionnaire was targeted to determine herd owners' awareness and practices for controlling ticks, acaricide preference, reasons for specific acaricide preference, and key sources/routes of acaricides. Moreover, information on seasonality of tick occurrence, frequency of acaricide treatment or application, the person in charge of treatment, the tick's response to treatment, trends in tick infestation, etc. were gathered. Oral approval was received from all study participants and the interviews were held at homestead either with male or female household.

### 2.5 Data management and analysis

The obtained data were entered into the Microsoft Office Excel 2010 computer program, and STATA version 20 software (Stata Corp., College Station, TX) was used for all statistical analyses. The proportions of variables that were recoded throughout the questionnaire survey were analyzed using descriptive statistics (frequencies and percentages). Simple logistic regression analysis via the R-software package was performed to see the association of respondent's knowledge and attitude towards tick control and prevention and its health and economic impact with gender. A 5% global significance level was used to carry out the tests.

### 2.6 Ethical consideration

The Southern Agricultural Research Institute's (SARI) Research Review Committee gave this study its ethical seal of approval after carefully reviewing it. Additionally, after outlining the goals and significance of the study and assuring the confidentiality of the information collected from the participant herdsmen, mutual agreement was gained from each of the interviewee. The study's participants gave their consent to participate, and they have granted themselves the right to decline the interview at any time.

## 3 Result

**Fig. 2:**
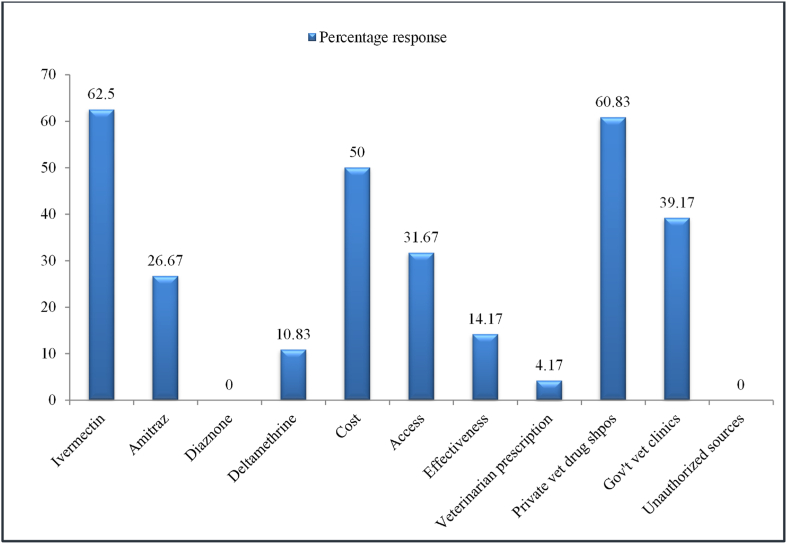
Pastoralist's acaricide preference, preference criterion and main acaricide route/sources.

Ivermectin is the most desired acaricide by 62.50% of the herd owners in the study area who were questioned due to its low cost in comparison to other acaricides since their preference for acaricides is primarily based on the chemical's price (Fig. 2). The primary source of acaricides in the area, according to the herd owners (60.83%), is private veterinary medicine stores, followed by public veterinary clinics (39.17%) (Fig. 2).

The majority of responders (65.83%) are aware of the diseases that tick infestations can spread to their animals. They identified the primary consequence of tick infestation as bacterial infection of the udder in nursing cows. The frequency of acaricide administration and/or treatment on their animals is based on the degree of tick infestation seen on the herd, according to 50.83% of research participants. Majority (60%) of the herdsmen relay on information delivered by vet drug shop seller about how to use acaricide on their animal. A total of 95.83% of respondents admitted they haven't received any instruction on how to apply acaricide on their pets (Table 1). All the respondents (100%) revealed that they didn't have the practice of measuring the dose of acaricide and animal's body weight prior to acaricide injection and/or application. As 72.22% our interviewees replied, herd owners themselves were dominantly responsible for application/treatment of acaricides on infested animals followed by CAHWs (27.77%).

**Table 1:** Herd owner's response on habit of acaricide usage, post-treatment response and trends of tick infestation in the study area.

Description of interview	Response	Frequency (%)
Knowledge on disease transmitted by ticks	Yes	79 (65.83)
	No	41 (34.17)
Frequency of acaricide usage	Once per month	12 (10.00)
	Once per two months	24 (19.17)
	Once per three months	23 (19.17)
	Depends on infestation	61 (50.83)
Source of information on how to use acaricides	Veterinarian	12 (10.00)
	Drug seller	72 (60.00)
	CAHWs	16 (13.33)
	Neighbor	20 (16.67)
Training on acaricide usage	Yes	5 (4.17)
	No	115 (95.83)
Practice of measuring acaricide dose	Yes	0 (0.00)
	No	120 (100.00)
Measuring animal's body weight before treatment	Yes	0 (0.00)
	No	120 (100.00)
Person responsible for acaricide treatment/application	Herd owner	87 (72.50)
	Veterinarian	0 (0.00)
	CAHWs	33 (27.50)

**Fig. 3:**
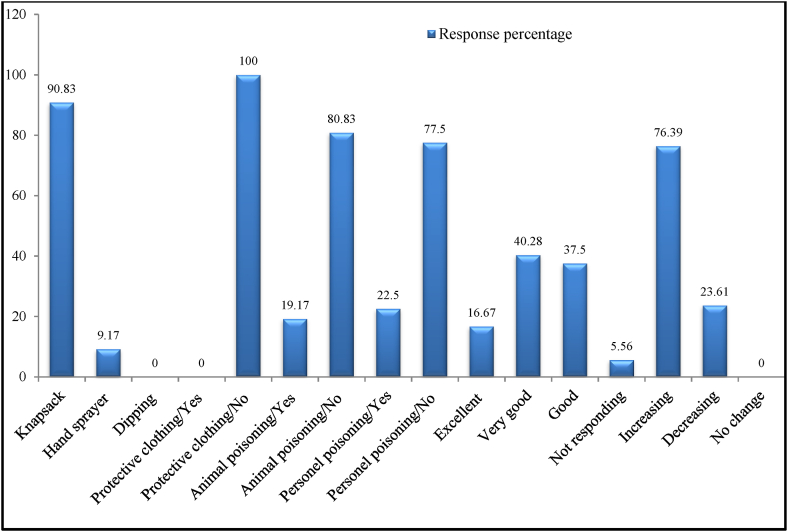
Pastoralists' response on methods of acaricide application, acaricide poisoning, response to acaricide treatment and current trends of tick infestation.

The majority (90.83%) of respondent herdsmen utilized the knapsack spraying technique to apply acaricide solutions. 100% of those questioned said that no protective clothing was ever worn when applying acaricide to tick-infested herds. Acaricide poisoning symptoms, which include scratching and restlessness in animals right after treatment, were reported by 19.17% of respondents to have happened to their treated animals (Fig. 3). Acaricide toxicity was also reported by 22.50% of respondents who had come into contact with acaricide chemicals. Response to acaricide treatment as demonstrated by a drop in the number of infested ticks was rated as very good, good, and excellent by 40.28%, 37.50%, and 16.67% of the respondents. The majority of our herdsmen who were contacted (76.39%) responded that their herd's tick infestation was on the rise compared to earlier time (Fig. 3).

According to knowledge score, 65% of the respondents have good knowledge about the health (both animal and human) and economic impact of tick infestation but their knowledge on acaricide usage is very poor. Majority (84.17%) of the respondents have positive attitude towards tick control campaign that has been conducted in the area. However, 15.83%) of the respondents have unfordable attitude as they are reluctant to tick control and consider tick as non-health threat to their herd (Table 2). However, practice score of the respondents revealed that majority (68.33%) of the respondents have poor practice of acaricide usage based on the criterion used to test their practice (Table 2).

**Table 2:** Mean knowledge, attitude and practice score of respondents towards acaricide usage and tick control.

KAP	Number of respondents	Category	Mean score
Knowledge	N = 120	Good	78 (65%)
		Poor	42 (35%)
Attitude	N = 120	Positive	101 (84.17%)
		Negative	19 (15.83%)
Practice	N = 120	Good	38 (31.67%)
		Poor	82 (68.33%)

Simple logistic regression analysis showed that male herd owners were 5.09 times (OR 95% CI = 2.30–11.72) more likely to have adequate knowledge towards the health and economic impact of ticks than female herdsmen. Similarly, herders who use acaricides rotationally were 3.22 times (OR 95% CI = 1.41–7.64) more likely to have adequate knowledge than those who use one type of acaricide on their herd. Moreover, herd owners who allow trained personnel (veterinarian, vet clinician or CAHWs) to apply acaricide on their herd were 2.66 times more likely have adequate knowledge towards acaricide usage than herders who apply acaricides by themselves (Table 3).

**Table 3:** Association of knowledge score of respondents with socio-demographic variables.

Variable	Category	N	Knowledge score		Simple logistic regression		
			Adequate	Inadequate	OR	OR 95% CI	*P*-Value
Gender	Male	70	56 (80%)	14 (20%)	5.09	2.30–11.72	0.0000000**
	Female	50	22 (44%)	28 (56%)			
Age	18–30 yrs	20	15 (75%)	5 (25%)	1.2	0.39–4.18	0.76
	31–40 yrs	44	34 (77.27%)	10 (22.72%)	1.36	0.55–3.47	0.51
	>40 yrs	56	40 (71.43%)	16 (28.57%)			
Marital status	Married	102	72 (70.58%)	30 (29.42%)			
	Not married	18	13 (72.22%)	5 (27.78%)	1.2	0.37–3.61	0.88
Herd type	Mixed	98	80 (81.63%)	18 (18.37%)			
	Single	22	18 (81.81%)	4 (18.19%)	1.01	0.33–3.82	0.98
Rotating acaricide	Yes	32	23 (71.87%)	9 (28.12%)	3.22	1.41–7.64	0.006**
	No	88	30 (34.09%)	58 (65.91)			
Frequency of acaricide application/year	<5 times	35	15 (42.85%)	20 (57.14%)			
	5-10 times	65	27 (41.54%)	38 (58.46%)	0.94	0.41–2.19	0.89
	>10 times	20	8 (40%)	12 (60%)	0.88	0.28–2.71	0.83
Who apply/inject acaricide	Herdsmen	87	27 (30.03%)	60 (68.96%)			
	Trained personnel	33	18 (54.54%)	15 (45.45%)	2.66	1.18–6.15	0.02**

N= Number of respondents, OR= Odds Ratio, CI= Confidence Interval, ** = statistically significant, herd type (mixed = having both large and small ruminants, single = having either large or small ruminants), trained personnel = veterinarian or vet clinician or trained CAHWs (Community Animal Health Workers).

According to this study there was statistically significant difference (P < 0.05) in attitude score between acaricide rotating and non-rotating herd owners. Consequently, herd owners who use acaricide rotationally were 3.20 times (OR 95% CI = 1.39–7.53) more likely to have positive attitude towards tick control than non-rotating ones (Table 4). Also, herder's preferences of person responsible to apply acaricide on their herd also have significant association (P < 0.05) with attitude score. Accordingly, herders who use trained individuals to apply acaricide were 6.61 times (OR 95% CI = 2.78–16.93) more positive to participate on tick control programs (Table 4).

**Table 4:** Association of attitude score of respondents with socio-demographic variables.

Variable	Category	N	Attitude score		Simple logistic regression		
			Positive	Negative	OR	OR 95% CI	*P*-Value
Gender	Male	70	60 (85.71%)	10 (14.29%)	1.32	0.48–3.55	0.58
	Female	50	41 (82.00%)	9 (18.00%)			
Age	18–30 yrs	20	14 (70.00%)	6 (30.00%)	0.33	0.09–1.18	0.08
	31–40 yrs	44	38 (86.36%)	6 (13.64%)	0.91	0.28–3.02	0.86
	>40 yrs	56	49 (87.50%)	7 (12.50%)			
Marital status	Married	102	68 (66.67%)	34 (33.33%)			
	Not married	18	10 (55.56%)	8 (44.44%)	0.63	0.23–1.77	0.36
Herd type	Mixed	98	70 (71.43%)	28 (28.57%)			
	Single	22	15 (68.18%)	7 (31.82%)	0.86	0.32–2.45	0.76
Rotating acaricide	Yes	32	17 (53.12%)	15 (46.88%)	3.20	1.39–7.53	0.007**
	No	88	23 (26.14%)	65 (73.86%)			
Frequency of acaricide application/year	<5 times	35	18 (51.42%)	17 (48.57%)			
	5-10 times	65	30 (46.15%)	35 (53.48%)	0.81	0.35–1.85	0.62
	>10 times	20	9 (45.00%)	11 (55.00%)	0.77	0.25–2.32	0.65
Who apply/inject acaricide	Herdsmen	87	25 (28.73%)	62 (71.27%)			
	Trained personnel	33	24 (72.72%)	9 (27.28%)	6.61	2.78–16.93	0.00000***

N= Number of respondents, OR= Odds Ratio, CI= Confidence Interval, ** = statistically significant, herd type (mixed = having both large and small ruminants, single = having either large or small ruminants), trained personnel = veterinarian or vet clinician or trained CAHWs (Community Animal Health Workers).

Similarly, the logistic regression analysis revealed that herders’ acaricide rotation practice and their preference of individuals to apply acaricide on their herd were significantly associated with practice score. Thus, acaricide rotating individuals were 5.31 times (OR 95% CI = 5.31) more likely to have good practice than non-rotating herders. On the same manner, herders who prefer trained individual for acaricide application were 7.21 times (OR 95% CI = 3.03–17.99) better in acaricide usage practices than herders who apply acaricide by themselves (Table 5).

**Table 5:** Association of practice score of respondents with socio-demographic variables.

Variable	Category	N	Practice score		Simple logistic regression		
			Good	Poor	OR	OR 95% CI	*P*-Value
Gender	Male	70	22 (31.43%)	48 (68.57%)	0.97	0.45–2.14	0.95
	Female	50	16 (32.00%)	34 (68.00%)			
Age	18–30 yrs	20	7 (35.00%)	13 (65.00%)	1.14	0.37–3.29	0.82
	31–40 yrs	44	13 (29.54%)	31 (70.54%)	0.88	0.37–2.07	0.78
	>40 yrs	56	18 (32.14%)	38 (67.86%)			
Marital status	Married	102	33 (32.35%)	69 (67.65%)			
	Not married	18	5 (27.78%)	13 (72.22%)	0.80	0.24–2.33	0.70
Herd type	Mixed	98	31 (31.63%)	67 (68.37%)			
	Single	22	7 (31.82%)	15 (68.18%)	1.00	0.35–2.65	0.98
Rotating acaricide	Yes	32	19 (59.38%)	13 (40.62%)	5.31	2.26–12.96	0.00016**
	No	88	19 (21.59%)	69 (70.41%)			
Frequency of acaricide application/year	<5 times	35	10 (28.57%)	25 (71.43%)			
	5-10 times	65	22 (33.85%)	43 (66.15%)	1.28	0.53–3.22	0.59
	>10 times	20	6 (30.00%)	14 (70.00%)	1.07	0.31–3.54	0.91
Who apply/inject acaricide	Herdsmen	87	17 (19.54%)	70 (80.46%)			
	Trained personnel	33	21 (63.64%)	12 (36.36%)	7.21	3.03–17.99	0.000000***

N= Number of respondents, OR= Odds Ratio, CI= Confidence Interval, ** = statistically significant, herd type (mixed = having both large and small ruminants, single = having either large or small ruminants), trained personnel = veterinarian or vet clinician or trained CAHWs (Community Animal Health Workers).

## 4 Discussion

In our study area, ivermectin was the most chosen acaricide, followed by amitraz and deltamethrin. However, studies by Kenyan [10] and Ugandan [3] researchers; respectively, found that the Amitraz group of acaricides was the most popular treatment for tick control. The majority of respondents said that their preference for an acaricide was determined by the chemical's market price because cheap acaricides are preferred to expensive ones. Conversely, according to Mugambi et al. [10], farmers in his research area preferred certain acaricides depending on their efficacy. In the current study area, private veterinary pharmacy stores were the primary supplier of acaricides, which were consistent with Ocaido et al.’ [11] findings in Uganda. It contrasted, however, with Moyo and Masika [12], who claimed that a government veterinary clinic served as the primary source of acaricide for farmers in South Africa's Eastern Cape Province.

The majority of our respondents answered that they are aware of diseases associated with tick infestation on their herd, despite the fact that they associate it with ticks' blood sucking process, such as teat wounding, bacterial complication, and body condition loss rather than other economically important tick borne diseases (TBD). According to a survey done in the Eastern Cape Province of South Africa [13], herdsmen stated gall sickness as the most common disease linked to tick infestation in their herd, which is in line with our finding. Similar to this, the majority of survey participants from Zimbabwe could identify tick diseases by their names or clinical and post-mortem symptoms such as cowdriosis, mastitis, anaplasmosis, body damage, babesiosis, and poor body condition [14]. However, investigations from Burkina Faso and Benin revealed that herd owners had very little knowledge about diseases spread by ticks and its associated problems [15]. The frequency of acaricide application in our current study area depended on the level of tick infestation. However, findings from Uganda [16] and Cameroon [17] indicated acaricides application of once a week by herd owners. Additionally, farmers in South Africa treat tick-infested cattle with acaricides every two weeks during the summer and winter months of the year [13].

Our interviewee stated that they simply guessed the volume of the acaricide rather than measuring the dosage (whether sprayed or injected) before treatment. Herders produce an aqueous formulation of an acaricide without consulting the manufacturer, just determining whether the formulation is adequate to wet the quantity of animals being treated. This might be as a result of a lack of specialists with the necessary training or a pastoralist's unwillingness to learn how to handle acaricides properly. However, Mugabi et al. [18] from Central Uganda hypothesized that the majority of the farmers interviewed knew the proper acaricide dilutions as advised by the manufacturers but chose to disregard them. In addition, according to Mugabi et al. [18] and Adehan et al. [19] from Uganda and Benin, respectively, farmers occasionally produce their own cost-efficient dilution of acaricides by mixing up different acaricide groups, which is thought to be more effective.

All of the respondents asserted that they had never measured animal body weight before acaricide medication, particularly injectable acaricide. This may be explained by the herdsmen's poor level of education (they are largely illiterate) and the district's lack of assistance for them. The majority of acaricides in the Bena-Tsemay district were applied on afflicted animals by herd owners (72.22%), followed by community animal health workers (CAHWs), and only sometimes by veterinarians and other qualified animal health professionals. This can be because pastoral areas have poor infrastructure and enormous physical sizes, making it challenging to provide standard fixed-point service. The application/treatment of acaricides by herders and CAHWs may also be due to a lack of qualified professionals (veterinarians and animal health specialists). On the other hand, according to surveys by Peeling and Holden [20], 82%, 88%, and 71% of respondents respectively, from Kenya, the Philippines, and Tanzania preferred CAHWs above other veterinary service providers.

In our study area, hand spraying with a backpack sprayer is the only way to provide acaricide to animals with tick infestations. The prohibitive cost of additional acaricide spraying techniques including dip washing, spray racing, and powered pumps may have prevented their usage. According to findings of [3,12,21,22], hand spraying was the most typical way for small-scale farmers to apply acaricides to their herd. However, the hand spraying methodology, according to these authors, is the least efficient and encourages the development of parasite resistance. On the other hand, the usage of hand treatment and spray race were identified as the most typical and economical method of acaricide application in Bhutan [23] and South-Western Uganda [24].

None of the herdsmen in our study donned protective clothing while applying acaricide to their herd, despite a few extremely small incidents of herdsmen being poisoned by acaricide. This suggests that herdsmen were regularly exposed to an acaricide solution, whether or not acaricide poisoning symptoms appeared. This study recommends caution and the wearing of protective clothing while using acaricides since long-term exposure to acaricide solutions may be hazardous and the health hazards connected with it are potentially fatal, according to earlier studies [25]. Small groups of survey respondents also mentioned temporary signs of acaricide poisoning in animals, suggesting that care should be taken when using acaricide on herds of tick-infested animals. As a result, animals with wounds and animals in very poor physical condition should be kept out of the group.

According to our study's participants, the tendency of tick infestation was thought to occasionally be on the rise. Because there is a lack of grazing rangeland, diverse animal herds are crowded together at grazing areas, which increases the spread of ticks from infested to uninfested animals. This was in agreement with Nasirian [26], who said that the global resultant trend of tick infestations in domestic ruminant groups has exhibited primarily an increasing trend globally over the past decades. This suggests that control measures to prevent tick infestations in domestic ruminant groups have not been successful. Similar to this, researchers have looked into a number of factors that are directly contributing to the global rise in tick infestation, including climatic change [27,28,29] and other ecological changes like habitat connectivity [30] and changes in human land management [31]. Additionally, scholars hypothesized that an increase in tick infestation on domestic animals may be caused by the destruction of wildlife habitat brought on by forest fragmentation, which increased interactions between wild animals roaming in urban and semi-urban areas and domesticated animals, leading to an increase in tick species picking [30]. The global annual and periodic patterns of tick infestation rates in humans, however, have shown declining trends over the past decades, demonstrating the preventative actions to ban human tick infestation have been successful, in contrast to increased tick infestation in domestic animals [32].

The observation that men had good knowledge than female could be due that the men get more opportunities to attend government initiated meetings and training programs held in the community. The Bena and Tsemay communities' culture also forbids women from attending public meetings and favors men instead, who are seen as the representatives of their families. Herd owners who rotate acaricides had adequate knowledge about acaricide usage. According to our study, most of acaricide rotating herdsmen are those individuals who allow trained individuals (vet clinicians and CAWHs) to treat their herd which in turn makes them to understand better about acaricide usage. However, majority of herdsmen in our study area lack adequate information about acaricide usage due to absence of trained individuals in their proximity. Poor education level of herdsmen and lack of extension services to deliver information play vital role in inadequate knowledge level of pastoralists and agro-pastoralists [33].

Strong association of acaricide rotation practice of herdsmen with their attitude and practice score of acaricide usage might be due the belief of herdsmen that rotating acaricide could bring better tick control on their herd. However, it is important to recognize those herdsmen with negative attitude and poor practice of acaricide usage will become good practitioner if they get awareness on acaricide usage. In most developing countries, herd owners obtain information about acaricide usage from knowledge poor individuals and develop malpractices of acaricide usage. In our current study area, rural vet drug shop attendants who lack technical knowledge are the main information node for farmers which in turn contribute for poor practice of acaricide usage. Similarly, previous study Kenya [10] stated that most of the herd owners obtain information about acaricide usage from vet shop attendant who are not trained in animal health care to properly advise farmers which leads the herd owners to malicious practice.

## 5 Conclusion and recommendations

According to this study, tick infestation is one of the biggest problems for small ruminant rearing practice in the area. Cost dependent acaricide preference from private vet drug shops was dominantly practiced in the study area. In addition to the main source of acaricide, private vet drug shops act as source of misleading information for herdsmen by untrained sellers which lead to acaricide usage malpractice. Lack of awareness creation training, lack of acaricide dosage measurement and animal body weight estimation and injection of acaricides by illiterate herdsmen were indicatives of poor acaricide usage practice in the area. The knowledge score of the respondents was significantly associated with the gender, acaricide rotation practice and herdsmen's personnel preference for acaricide application on their herd. Although it was practiced by small portion of our interviewed herdsmen, rotation of acaricide and preference of trained technicians to apply acaricide chemical were significantly interlinked with practice and attitude score of the respondents. In conclusion, awareness campaigns should be targeted to minimize/avoid malpractices due to knowledge, attitude and practice gaps which lead to ineffective acaricide usage. Additionally, the public should be made aware of the potential health risks linked to tick infestation as well as the need for staff safety when using acaricides on infected animals and elsewhere.

## 6 Study limitations

It was challenging to incorporate all the estimated respondents because of the many difficulties encountered during our interview process (household displacement due to natural disasters, pastoralists' mobility, and unwillingness of individuals to engage in the study). This study was designed to specific locations (pastoral and agro-pastoral villages), therefore, the findings cannot be generalized to other areas with different type of farming practices. The questionnaire was written in English, but the interview was conducted in a regional dialect, so there may have been some bias as a result of the language and cultural context. However, we were able to minimize this potential bias because the enumerators were familiar with the dialect, culture, and farming practices of the farmers interviewed.

## Author contribution statement

Tegegn Tesfaye, Aschenaki Abate: conceptualization, designing study methodology, field data collection, questionnaire development, local language translation, data analysis and interpretation, review writing and original draft writing.

## Funding statement

This research's fund was obtained from Southern Nations Nationalities and People Regional (SNNPR) Government of Ethiopia; budgeted for regional research activities conducted under Southern Agricultural Research Institute (SARI).

## Data availability statement

Data will be made available on request.

## Declaration of competing interest

The authors declare that they have no known competing financial interests or personal relationships that could have appeared to influence the work reported in this paper.
